# Congenital Pulmonary Alveolar Proteinosis

**DOI:** 10.1155/2013/764216

**Published:** 2013-04-27

**Authors:** Saber Hammami, Khaled Harrathi, Khaled Lajmi, Samir Hadded, Chebil Ben Meriem, Mohamed Néji Guédiche

**Affiliations:** ^1^Pediatrics Department, University of Monastir, Faculty of Medicine, Monastir 5000, Tunisia; ^2^ORL Department, University of Monastir, Faculty of Medicine, Monastir 5000, Tunisia

## Abstract

Pulmonary alveolar proteinosis (PAP) is a rare syndrome characterized by pulmonary surfactant accumulation within the alveolar spaces. It occurs with a reported prevalence of 0.1 per 100,000 individuals. Two clinically different pediatric types have been defined as congenital PAP which is fatal and a late-onset PAP which is similar to the adult form and less severe. The clinical course of PAP is variable, ranging from spontaneous remission to respiratory failure. Whole-lung lavage is the current standard treatment for PAP patients. We report a new congenital case of PAP.

## 1. Introduction

Pulmonary alveolar proteinosis (PAP) is rare respiratory disease characterized by the accumulation of surfactant-derived material in the lungs [1, 2]. In pediatric practice, two forms are recognized: congenital alveolar proteinosis (CAP) and a later-onset form that is generally less severe. The main symptoms are nonspecific. Bronchoalveolar lavage is the key to diagnosis [3]. We present a new pediatric case of congenital alveolar proteinosis.

## 2. Case Report

**Figure 1:**
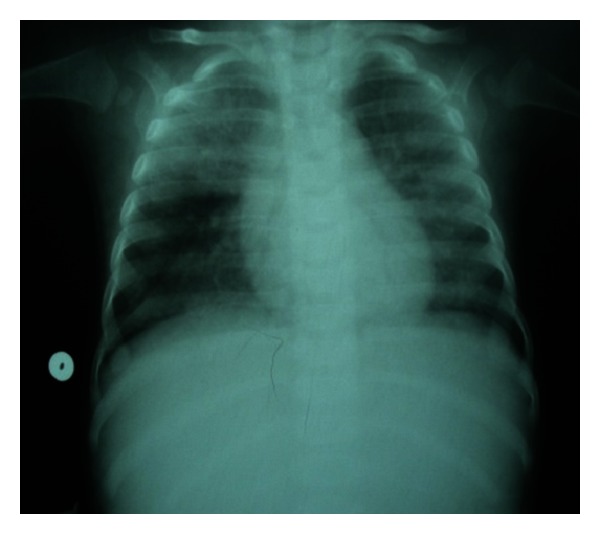
Chest X-ray: bilateral alveolointerstitial infiltrate.

**Figure 2:**
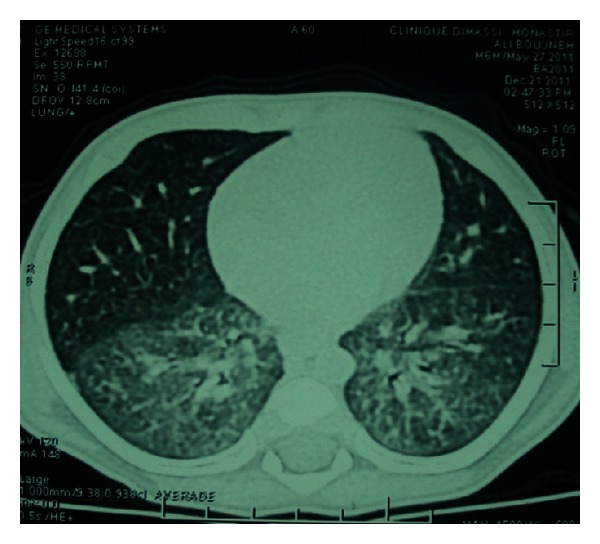
Chest computed tomography: bilateral interstitial infiltrate.

A 3-month-old boy was referred to our pediatrics department because of chronic tachypnea and weight loss. The patient was born at term in 2011, with no immediate postnatal respiratory distress. The parents were second-degree consanguineous. The infant presented with a 3-week history of dyspnea and oxygen dependence. The infant had a sister who had died at the age of 3 months from respiratory failure. Physical examination revealed that he weighed 4.5 kg and his height was 58 cm. His heart rate was 130 beats per minute and his respiratory rate was 60 breaths per minute. He had peripheral cyanosis and intercostal retractions. Fine inspiratory crackles could be heard throughout the chest on auscultation. His transcutaneous oxygen saturation while breathing room air was 80%. The minimum oxygen flow needed to have transcutaneous oxygen saturation greater than 92% was 8 L/mn. The liver and spleen were not enlarged. Laboratory tests showed low total protein 54 g/L and albumin 20 g/L, liver enzymes, renal function, and full blood count were normal. Inflammatory markers were slightly increased. Arterial blood gases on oxygen were 7.38, Pco2 4.81 KPa, and Po2 8.53 KPa. Chest X-ray revealed diffuse alveolar infiltration with an air bronchogram (Figure 1). Chest CT scan showed diffuse interstitial and alveolar infiltration (Figure 2). Echocardiography was normal. Respiratory fungal, viral, and bacterial pathogens were all negative. HIV testing was also negative. Because of a typical chest computed tomography and chest X-ray, and after exclusion of metabolic or infectious causes, PAP was suspected. A bronchoalveolar lavage was performed. Macroscopic appearance and light microscopy of bronchoalveolar lavage fluid stained with PAS showed extracellular positive proteinaceous material and lipid-laden macrophages, consistent with the diagnosis of PAP. The aspirated fluid was reported to be clear and a silver stain was negative for *Pneumocystis jiroveci*. Three lung lavages were performed for treatment. Initially,  the infant improved with less dyspnea and cough. Secondary, evolution was unfavorable. The infant died of uncontrollable respiratory failure.

## 3. Discussion

PAP is rare in children, with only a few dozen cases reported in the literature [1]. PAP is characterized by the intra-alveolar accumulation of surfactant lipids and proteins impairing gas exchange and resulting in progressive respiratory insufficiency. Today, PAP can be categorized into three different classes: congenital, acquired, and secondary to other conditions, such as malignancy (i.e., leukemia and lymphoma) and immunodeficiency [4, 5]. Two forms are encountered in pediatric practice: congenital alveolar proteinosis (CAP) and a later-onset form that is generally less severe. These 2 types differ in respect to etiology, clinical course, therapy, and outcome [2]. Family history of a similarly affected sibling or consanguinity suggests autosomal recessive transmission [1]. In our case, infant had history of both similarly affected sister and consanguineous parents. The later-onset form always appears after a postnatal, symptom-free period ranging from a few weeks to several years. A clinical diagnosis of PAP can often be made by the characteristic milky colour of the bronchoalveolar lavage fluid. In our case, the initial tracheobronchial lavage was described as a bronchoalveolar lavage with a clear return [1]. The main symptoms are non-specific, including progressive-onset dyspnea during feeding or exercise and then at rest, cough, cyanosis, and digital clubbing. Asthenia and growth retardation are common. Our case had respiratory difficulty and poor growth. The progression of the disease is very variable, ranging from asymptomatic forms diagnosed with chance to early-onset forms that progress rapidly and result in uncontrollable respiratory failure [1, 6]. In our case, evolution was rapidly fatal and the infant died of uncontrollable respiratory failure. The classic radiologic appearance of PAP is bilateral, symmetric, and perihilar airspace consolidation in a bat-wing distribution [2]. Whole-lung lavage has been widely used as a treatment and has been associated with long-term survival. This method is still the only therapy that is really effective. In our case, three whole-lung lavages had been performed with initial improvement [7]. Other therapeutic trials have been proposed such as: lung transplantation, administration of GM-CSF, and gene-therapy [1]. 

## 4. Conclusion

Since symptoms of PAP are not specific, missed and delayed diagnoses are more frequent. In fact, diagnosis of postnatal onset of PAP should be considered in every infant with persistent respiratory distress and poor growth with diffuse alveolar and interstitial infiltrate. History of similarly affected sibling or consanguineous parents strongly suggests diagnosis of PAP.
